# The effect of Fasting during Ramadan on the Kidney functions of Stage III-IV Chronic Kidney Disease Patients

**DOI:** 10.12669/pjms.37.4.3661

**Published:** 2021

**Authors:** Ahmet Karatas, Ebru Canakci, Yeliz Kasko Arici, Mervegul Kaya, Beyza Sayim

**Affiliations:** 1 Dr. Ahmet Karatas, MD. Nephrologist, Associated Professor, Department of Internal Medicine, Division of Neprology, Education and Research Hospital, Ordu University School of Medicine, Ordu, Turkey; 2 Dr. Ebru Canakci, MD, Anesthesiologist, Associate Professor, Department of Anesthesiology and Reanimation, Education and Research Hospital, Ordu University School of Medicine, Ordu, Turkey; 3 Dr. Yeliz Kasko Arici, PhD. Biostatistics Specialist, Assistant Professor, Department of Biostatistics and Medical Informatics, Ordu University School of Medicine, Ordu, Turkey; 4 Mervegul Kaya, MD, Family Medicine Specialist, Attending Physician, Department of Family Medicine, Education and Research Hospital, Ordu University School of Medicine, Ordu, Turkey; 5 Beyza Sayim, MD, Researcher Fellow Department of Internal Medicine, Education and Research Hospital, Ordu University School of Medicine, Ordu, Turkey

**Keywords:** Ramadan fasting, Chronic kidney disease, Metabolic parameters

## Abstract

**Objectives:**

Examine the effect of fasting during Ramadan on kidney functions in patients with chronic kidney disease.

**Methods:**

The study was conducted on 130 patients with stage III-IV chronic kidney disease (CKD), who were admitted to the Ordu University nephrology polyclinic during the month before Ramadan and one month after Ramadan in 2019. Blood samples were taken in the morning after 12 hours of fasting.

**Results:**

There was a statistically significant difference between BUN in the fasting group before and after the month of Ramadan. The median BUN before Ramadan was 26.65 mg/dl, the median after Ramadan was 24.05 mg/dl (p=0.004).There was a statistically significant difference between the nonfasting groups before and after Ramadan with respect to creatinine level. Median creatinine before Ramadan was 1.69 mg/dl,and the median after Ramadan was 1.86 mg/dl (p <0.001).There was a statistically significant difference between the fasting groups before and after Ramadan with respect to creatinine levels. Fasting group ,the median before Ramadan was 1.5 mg/dl, and the median after Ramadan was 1.42 mg/dl (p = 0.038).The impact of independent variable of fasting, using linear regression was found to be statistically significant (p_post-_<0.001). The eGFR was 14.826 points higher in those who fasted after Ramadan than in those who did not.

**Conclusion:**

Fasting during the month of Ramadan does not deteriorate kidney functions and even leads to a moderate improvement in kidney functions. Taking these results into consideration, fasting may be advised for patients with stage III-IV CKD who want to fast and remain in stable condition.

## INTRODUCTION

Fasting from dawn to dusk during the holy month of Ramadan has been prescribed to all Muslims.^1^ Even though many studies suggest that fasting during Ramadan does not have a negative impact on healthy people, contradictory results were found by a few studies were conducted on the effect of Ramadan in patients with stage III-IV CKD.^2^-^5^ However, there are no guidelines or standard protocols related to this issue. For these reasons, prohibiting or dissuading a patient with CKD from fasting during Ramadan is controversial.^6^

Ramadan may lead to complications such as dehydration, low blood pressure and hyperviscosity, all of which cause more kidney damage and susceptibility to thrombosis in patients with chronic kidney disease.^7^ Fasting during Ramadan has varying impacts on inhibitory factors contributing to urinary calculus formation, urinary calcium excretion and urine precipitate concentrations.^8^

Some studies have suggested a temporary improvement in the estimated glomerular filtration rate (E-GFR) and the decline in proteinuria in patients with CKD or kidney transplant.^9^ In our study, we looked at the effect of fasting during Ramadan on kidney functions in patients with chronic non dialysis kidney disease.

## METHODS

To perform our study, ethical approval was obtained (No 2018/114 dated 24.05.2018) from the Ordu University Clinical Research Ethics Committee. It included 130 patients with stage III-IV CKD admitted to the Ordu University. Patients with malignancies, advanced heart failure, acute infection, diabetes mellitus using insulin as well as patients on dialysis and recipients of kidney transplants were excluded. The diagnosis of dialysis CKD was established according to the KDIGO guideline.^10^ The estimated glomerular filtration rate (eGFR) value of the patients was calculated using the Kidney Disease Epidemiology Collaboration creatinine equation.^11^

Complete blood count was examined using the CELL-DYN RUBY (Abbott, IL, USA) device. Routine biochemical analysis were measured using the COBAS c501 (Roche, Basel, Switzerland) module. BMI was computed in all patients (BMI = weight (kg)/height (m^2^)).

### Statistical Methods

The data were analyzed using IBM SPSS program v23. Normal distribution was determined using the Kolmogorov-Smirnov test. The chi-square test was used to compare categorical variables according to the groups. To analyze changes over time, two paired-sample t-tests were used for normally distributed data and the Wilcoxon test was used for non normally distributed data. Linear regression analysis was used to analyze the independent variables that affect the eGFR. The level of significance was set at p<0.050.

## RESULTS

There was no statistically significant difference between the groups with respect to gender. A total of 63.6% of those who did not fast were men, and 36.4% were women; 62.5% of those who fasted were men, and 37.5% were women. The categorical variables for the groups are presented in Table-I. The features of the quantitative variables that belong to the demographic characteristics of the groups are displayed in Table-II.

**Table-I:** Comparison of the categorical variables with respect to groups.

		Nonfasting n=66	Fasting n=64	Total n=130	p
Gender	Female	24 [36.4]	24[37.5]	48 [36.9]	0.893
	Male	42 [63.6]	40 [62.5]	82 [63.1]	

: Chi-square test statistics.

**Table-II:** Comparison of quantitative variables, which are demographic characteristics, based on groups.

		Nonfasting	Fasting	Total	p
Age	Mean ± Standard Deviation	65.38 ± 10.05	61.16 ± 14.78	63.3 ± 12.73	0.06
	Mean [Min. - Max.]	67 [23 - 78]	63 [26 - 90]	64.5 [23 - 90]	
BMI	Mean ± Standard Deviation	29.37 ± 4.04	29.2 ± 4.41	29.29 ± 4.21	0.820
	Mean[Min. - Max.]	28.25 [23.3 - 37.5]	29.05 [19.7 - 45.7]	28.5 [19.7 - 45.7]	

t: Statistics from independent two-sample t tests.

There was no statistically significant difference between groups with respect to mean ages (p=0.06) and with respect to average body mass index (p=0.820). The comparisons of kidney function tests and all biochemical parameters are presented in Tables-IIIa, IIIb.

**Table-IIIa:** Comparison of biochemical parameters between and within groups.

Parameter	Time		Nonfasting	Fasting	Total	p
Glucose(mg/dl)	Pre-Fasting	Mean ± Standard Deviation	141.89 ± 58.59	115.93 ± 27.73	129.11 ± 47.7	0.018
		Mean (Min. - Max.)	119 (68 - 369)	111 (81 - 253)	115 (68 - 369)	
	Post-Fasting	Mean ± Standard Deviation	141.3 ± 66.32	111.71 ± 24.61	126.73 ± 52.28	0.008
		Mean (Min. - Max.)	113 (77 - 407)	102.5 (86 - 191)	108.5 (77 - 407)	
	p		0.963	0.038		
BUN (mg/dl)	Pre-Fasting	Mean ± Standard Deviation	36.84 ± 22.61	30.66 ± 14.53	33.8 ± 19.25	0.124
		Mean (Min. - Max.)	27.45 (15.1 - 149)	26.65 (9 - 77.8)	27.1 (9 - 149)	
	Post-Fasting	Mean ± Standard Deviation	36.41 ± 18	25.51 ± 9.01	31.05 ± 15.26	< 0.001
		Mean (Min. - Max.)	29.75 (15.5 - 88)	24.05 (7.1 - 46.7)	26.95 (7.1 - 88)	
	p		0.405	0.004		
Creatinine (mg/dl)	Pre-Fasting	Mean ± Standard Deviation	2.08 ± 0.93	1.62 ± 0.55	1.86 ± 0.8	0.002
		Mean (Min. - Max.)	1.69 (1.1 - 4.8)	1.5 (0.6 - 3.5)	1.6 (0.6 - 4.8)	
	Post-Fasting	Mean ± Standard Deviation	2.29 ± 1.11	1.56 ± 0.55	1.93±0.95	< 0.001
		Mean (Min. - Max.)	1.86 (1.2 - 5.9)	1.42 (0.6 - 3.4)	1.65 (0.6 - 5.9)	
	p		<0.001	0,038		
e-E-GFR (ml/dk/1.73 m^2^)	Pre-Fasting	Mean ± Standard Deviation	35.79 ± 13.73	46.2 ± 17.82	40.91 ± 16.66	0.001
		Mean (Min. - Max.)	36.08 (11 - 67)	45.15 (14.6 - 124.2)	41.09 (11 - 124.2)	
	Post-Fasting	Mean ± Standard Deviation	33.29 ± 14.52	48.12 ± 18.55	40.59 ± 18.16	< 0.001
		Mean (Min. - Max.)	34.17 (8 - 89.1)	48.93 (17.6 - 134)	38.64 (8 - 134)	
	p		< 0.001	0.107		
LDL- cholesterol (mg/dl))	Pre-Fasting	Mean ± Standard Deviation	104.5 ± 37.3	108.11 ± 35.19	106,26 ± 36,19	0.573
		Mean (Min. - Max.)	100.5 (13.2 - 196.8)	108.2 (30 - 200.2)	106.4 (13.2 - 200.2)	
	Post-Fasting	Mean ± Standard Deviation	107.45 ± 32.01	111,29 ± 36,95	109.32 ± 34.43	0.529
		Mean (Min. - Max.)	104.1 (44.4 - 178.6)	112.2 (30 - 212.4)	105.6 (30 - 212.4)	
	p		0.350	0.249		
Potassium (mmol/l)	Pre-Fasting	Mean ± Standard Deviation	4.83 ± 0.55	4.67 ± 0.55	4.75 ± 0.55	0.115
		Mean(Min. - Max.)	4.87 (3.7 - 6.2)	4.71 (3.7 - 6.3)	4.78 (3.7 - 6.3)	
	Post-Fasting	Mean ± Standard Deviation	4.97 ± 0.57	4.81 ± 0.57	4.89 ± 0.58	0.096
		Mean(Min. - Max.)	5.01 (3.8 - 6.2)	4.84 (3.5 - 6.5)	4.88 (3.5 - 6.5)	
	p		0.044	0.064		

t: Statistics from in dependent two-sample t tests, U: Statistics from the Mann–Whitney U test, Z: Statistics from the Wilcoxon test, t: Statistics from paired two-sample t-tests.

**Table-IIIb:** Comparison of biochemical parameters between and within groups.

Parameter	Time		Non fasting	Fasting	Total	p
BCR (mg/mg)	Pre-Fasting	Mean ± Standard Deviation	17.9 ± 7.15	19.62 ± 9.74	18.75 ± 8.53	0.386
		Mean (Min. - Max.)	16 (8.6 - 49.37)	17.1 (8.74 - 66.24)	16.4(8.6-66.24)	
	Post-Fasting	Mean ± Standard Deviation	16.57 ± 6.32	17.19 ± 6.22	16.88 ± 6.25	0.376
		Mean (Min. - Max.)	14.9 (8.51 - 55)	15.66 (6.09 - 41.7)	15.51(6.09- 55)	
	p		0.177	0.054		
Total cholesterol (mg/dl)	Pre-Fasting	Mean ± Standard Deviation	190.57 ± 40.46	192.44 ± 47.71	191.49 ± 44.01	0.810
		Mean (Min. - Max.)	186.5 (103 - 307)	192.5 (86 - 324)	190.5 (86- 324)	
	Post-Fasting	Mean ± Standard Deviation	184.27 ± 40.19	191.92 ± 48.35	188.04 ± 44.39	0.328
		Mean (Min. - Max.)	181.5 (103 - 287)	194.5 (112 - 366)	188 (103 - 366)	
	p		0.101	0.878		
HDL- cholesterol (mg/dl)	Pre-Fasting	Mean ± Standard Deviation	45.48 ± 12.09	47.64 ± 12.64	46.54 ± 12.37	0.231
		Mean (Min. - Max.)	42 (29 - 79)	47.5 (27 - 91)	45 (27 - 91)	
	Post-Fasting	Mean ± Standard Deviation	42.68 ± 10.88	47.56 ± 12.57	45.08 ± 11.95	0.017
		Mean (Min. - Max.)	41 (20.8 - 79)	46.5 (27 - 82)	43 (20.8 - 82)	
	p		<0.001	0.820		
Triglyceride (mg/dl)	Pre-Fasting	Mean ± Standard Deviation	186.11 ± 85.59	151.59 ± 81.8	169.12 ± 85.2	0.013
		Mean (Min. - Max.)	166 (60 - 398)	134 (34 - 475)	148 (34 - 475)	
	Post-Fasting	Mean ± Standard Deviation	174.24 ± 74.12	150.23 ± 83.11	162.42 ± 79.29	0.016
	Mean (Min. - Max.)	161.5 (71 - 388)	123 (60 - 475)	148.5(60 - 475)		
	p		0.259	0.987		
UACR (mg/g)	Pre-Fasting	Mean ± Standard Deviation	1021.81 ± 2002.97	504.09 ± 987.45	766.93±1601.63	0.084
	Mean (Min. - Max.)	86.69 (0 - 9111.5)	26.6 (0 - 4535)	57.34(0-9111.5)		
	Post-Fasting	Mean ± Standard Deviation	954.35 ± 1819.72	440.32 ± 813.56	701.29±1434.69	0.119
	Mean (Min. - Max.)	119.55 (0 - 7184.4)	43.08 (0 - 3739)	72.1(0- 7184.4)		
	p		0.415	0.347		
HbA1c (%)	Pre-Fasting	Mean ± Standard Deviation	6.47 ± 1.35	5.88 ± 0.76	6.18 ± 1.14	0.016
		Mean (Min. - Max.)	6 (4.6 - 11.5)	5.7 (4.8 - 8.4)	5.75 (4.6- 11.5)	
	Post-Fasting	Mean ± Standard Deviation	6.49 ± 1.31	5.92 ± 0.81	6.21 ± 1.12	0.011
		Mean (Min. - Max.)	6.05 (4.8 - 11.5)	5.7 (4.8 - 8.7)	5.9 (4.8 - 11.5)	
	p		0.182	0.293		
Uric acid (mg/dl)	Pre-Fasting	Mean ± Standard Deviation	7.06 ± 1.42	6.64 ± 1.59	6.85 ± 1.51	0.119
		Mean (Min. - Max.)	7.05 (4.2 - 10.4)	6.4 (3.3 - 11.2)	6.8 (3.3 - 11.2)	
	Post-Fasting	Mean ± Standard Deviation	7.07 ± 1.3	6.36 ± 1.4	6.72 ± 1.39	0.003
		Mean (Min. - Max.)	7.15 (4.1 - 10.4)	6.25 (3.3 - 9.4)	6.8 (3.3 - 10.4)	
	p		0.909	0.021		

: Statistics from in dependent two-sample t tests, U: Statistics from the Mann-Whitney U test, Z: Statistics from the Wilcoxon test, t: Statistics from paired two-sample t-tests.

There was a statistically significant difference between fasting groups before and after Ramadan with respect to BUN. The median BUN before Ramadan was 26.65 mg/dl, and the median after Ramadan was 24.05 mg/dl(p=0.004). There was a statistically significant difference between the non fasting groups before and after Ramadan with respect to creatinine level. Median creatinine before Ramadan was 1.69 mg/dl, and the median after Ramadan was 1.86 mg/dl (p<0.001). Similarly statistically significant difference was seen between the fasting groups before and after Ramadan with respect to creatinine levels. The median before Ramadan was 1.5 mg/dl, and the median after Ramadan was 1.42 mg/dl (p =0.038).Moreover, there was statistically significant difference between the groups post-Ramadan with respect to median uric acid level and between fasting groups before and after Ramadan with respect to mean uric acid level. The median uric acid level pre-Ramadan was 6.64mg/dl, and the median post-Ramadan was 6.36 mg/dl (p=0.021).There was a statistically significant difference between the fasting groups before and after Ramadan with respect to glucose level (p=0.038).The median glucose level before Ramadan was 111mg/dl, and the median after Ramadan was 102.5 mg/dl and in pre-Ramadan groups with respect to median HbA1c. The median of those who did not fast was 6%, and the median of fasted was 5.7% (p=0.016). Statistically significant difference was also seen between the post-Ramadan groups with respect to median HbA1c. The median of those who did not fast was 6.05%, and the median of those who fasted was 5.7% (p=0.011).

**Fig.1:**
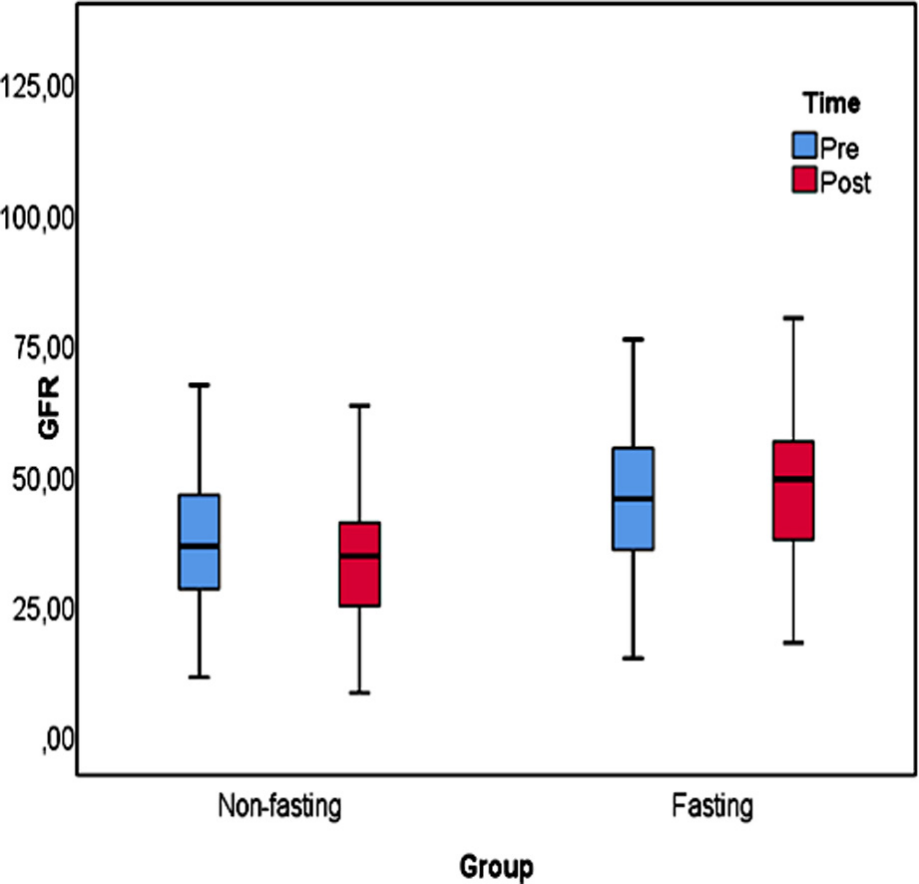
Box plot for E-GFR.

When the impact of the independent variable of fasting (which affects E-GFR post-Ramadan) was examined using linear regression, the established regression model was found to be statistically significant (F_post_=25.838,p_post_<0.001).The eGFR value of those who fasted after Ramadan was 14.826 points higher than that of those of who did not. When the impact of the independent variable of fasting (which affects eGFR pre-Ramadan) was examined using linear regression, the established regression model was found to be statistically significant (Fpre=13.978, pre<0.001).The eGFR value of those who fasted before Ramadan was 10.415 points higher than that of those of who did not. The results of our linear regression analysis are presented in Table-IV. eGFR values of fasting and fasting patients are presented graphically in Fig.1.

**Table-IV:** Examination of the relationship between E-GFR and fasting through linear regression analysis.

		Beta	Standard error	Standardized beta (95% CI)	p
Post-Ramadan	The Constant	33.293	2.046		< 0.001
	Fasting	14.826	2.917	0.41 (9.055 - 20.597)	< 0.001
Pre-Ramadan	The Constant	35.786	1.955		< 0.001
	Fasting	10.415	2.786	0.314 (4.903 - 15.926)	< 0.001

F_post-_= 25.838, p_post-_<0.001, R2= 0.168, Adjusted R2= 0.161, F_pre-_= 13.978, p_pre-_0.001, R2= 0.098, Adjusted R2= 0.091

## DISCUSSION

In our study, we showed that fasting during Ramadan did not worsen kidney function in patients with stage III-IV chronic kidney disease who are not under going dialysis; to the contrary, fasting positively affected kidney function.

The changes in the kidney functions of 15 chronic kidney disease patients who fasted during Ramadan (baseline eGFR<60 ml/min/1.73m^2^) were examined in a study in the literature. After the month of Ramadan, the changes in the baseline values of eGFR between the fasting and non-fasting groups were not significantly different. These findings suggest that eGFR did not change significantly in the patients with chronic kidney disease yet fasting during Ramadan may damage the kidney tubular cells of these patients.^5^

In a study by Bakhit et al, the authors found that fasting during Ramadan was associated with deterioration of kidney function in patients with Stage III or higher CKD, particularly in summer, when the weather was hot.^12^ In another study, which had been conducted to determine whether fasting during Ramadan affects kidney function in kidney transplant recipients with normal or impaired graft function, it was found that fasting during Ramadan was not associated with any significant adverse effects in kidney transplant recipients with normal or impaired graft function and that fasting during Ramadan was safe for these patients after one year.^13^

Kara et al. found that fasting during Ramadan was not associated with a reduced risk of renal function in patients with stage 3-5 CKD.^14^ In a study by Bernich et al., which was conducted in patients with stage 3-5 CKD, they found a considerable improvement in eGFR during the fasting month and the consecutive month.^9^ In our study, similar to the results of Bernich et al., we found that there was a significant improvement in eGFR after Ramadan compared to eGFR before Ramadan.

In the study conducted by NasrAllah and Osman, patients who fasted had a 30% increase in creatinine level and a deterioration in kidney function.^15^ In a study carried out by Bakhit et al., 33% of 65 patients with stage 3 CKD had an increase in creatinine levels after fasting compared to baseline, which in turn negatively affected kidney function.^12^ In the study of Abushady et al. in diabetic patients, creatinine levels increased in the group with diabetic nephropathy when compared to those without diabetic nephropathy.^16^ In contrast to these studies, another study found that fasting did not have a negative impact on creatinine levels.^17^ In the present study, there was a significant decrease in creatinine levels compared to basal values, suggesting that there is an improvement in kidney function in fasting CKD patients.

Emami-Naini et al. claimed that there was an increase, if not significantly, in BUN values after fasting. As a result of their studies, they emphasized the importance of hydration.^18^ In a study performed by Kara et al., it was found that there was no significant difference in urea values in stage 3-5 CKD patients, regardless of whether they were fasting.^14^ In the present study, we found a significant decrease in BUN values in stage 3-5 CKD fasting patients post fasting. Furthermore, we did not observe any difference in BUN/Creatinine ratio (BCR) pre- and post-fasting. Based on this finding, we conclude that fasting does not cause dehydration.

The urine albumin creatinine ratio (UACR) is a standard kidney damage test, in which protein (albumin) is abnormally excreted.^11^ In the study of El-Wakil et al., no increase in the excretion of urinary protein was observed post fasting.^5^ According to the study of Bernieh et al., changes in urinary protein excretion and changes in the UACR were not significant before, during or after Ramadan in fasting patients.^9^ Our studies are consistent with the literature in this respect.

Chowdhury et al. did not find any significant change in HbA1c levels in their study on stage 3 CKD and Type 2 DM patients.^19^ In the present study, we observed that there was a significant improvement in both glucose and HbA1c values in fasting patients. We believe that this result is because fasting has a positive impact on metabolism and that our patients follow regular eating habits and the recommendations of their physicians during the month of Ramadan.

In another study conducted by Kara et al., fasting did not affect potassium levels.^14^ Similarly, in the present study, there was no difference in potassium levels between fasting and non-fasting patients.^14^

Serum uric acid levels increase in chronic kidney failure. Because hyperuricemia might occur due to decreases in kidney function with CKD, hyperuricemia itself can cause chronic kidney failure and lead to progression of the disease.^20^ In a study by Erdem et al. on CKD patients, mean serum uric acid levels of patients with stage 3-5 predialysis chronic renal failure were higher than normal.^21^ In the study of Bakhit et al., it was claimed that fasting did not change uric acid levels.^12^ In the present study, fasting had a positive effect on uric acid levels. This situation was seen for the first time in the literature.

The effects of fasting on lipid metabolism are not clearly known. El-Wakil et al. found that fasting had no effect on the lipid metabolism of CKD patients.^5^ Chowdhury et al. did not observe any significant difference in lipid parameters after fasting (19).^19^ In our study, triglyceride levels in the fasting group decreased. We found that fasting did not have a negative effect on lipid metabolism, and even positively affected triglyceride levels in CKD patients with cardiovascular risk factors.

### Limitation of the Study

Our cases are from a single region. The absence of long-term results is another limitation of our study.

## CONCLUSION

Fasting of CKD patients during the month of Ramadan was well-tolerated and did not disrupt kidney functions in stage III-IV CKD patients. Contrary to popular belief, fasting during the month of Ramadan does not impair kidney functions and even leads to moderate improvement in kidney functions. The same situation is true for metabolic parameters. Considering these results, patients with stable stage III-IV CKD under close monitoring can be recommended to fast.

### Authors Contribution:

**AK:** Concept, Study Design, Literature review, Data collection, prepared the manuscript and is responsible for integrity of research.

**EC:** Literature review, Data collection, Final review, Study design, Data collection.

**YKA:** Conduct the study, Data collection and analysis.

**MK:** Conduct the study, Data collection and analysis.

**BS:** Literature Review, Data Collection, Final review.
